# Supplementary material for: A systems genomics approach uncovers molecular associates of RSV severity
Source: PLoS Comput Biol. 2021 Dec 28;17(12):e1009617. doi: 10.1371/journal.pcbi.1009617 (PMC8746750; doi:10.1371/journal.pcbi.1009617)
Supplement: S1 Text — Supplemental Materials and Methods and Tables A-G. The text and tables describe details of the data preprocessing and analysis as well as the demographics of the study cohort. (DOCX) [file pcbi.1009617.s001.docx]

S1 Text: Supplementary Materials and Methods

*Transcriptomic data preprocessing*

Four types of RNA-seq data, measuring the transcriptional profiles of nasal epithelial cells (NT) and three specific types of blood cells (CD4, CD8, and CD19), were used in this study. Their sequencing libraries were constructed using the NexteraXT library kit (Illumina, San Diego, CA) and then sequenced on the Illumina HiSeq2500 platform. Sequences were aligned against human genome version of hg19 using STARv2.5, counted with HTSeq, and normalized by Fragments Per Kilobase of transcript per Million mapped reads (FPKM). We winsorized potential outliers at the gene-level. Below we describe these preprocessing procedures in detail.

*Quality assurance analysis*

For all four types of data, we remove samples with less than 5 million total mapped reads and extremely low mean Pearson correlation with other samples (as determined by visual examination of the between-sample correlation heatmap). The remaining samples were used in subsequent analyses, and we summarize the results in Table S5.

*Non-specific filtering, winsorization, and univariate screening*

We applied several filtering procedures based on both maximum expression values and inter-quartile range (IQR) to identify subsets of genes for further investigation.

1. First, we computed $M_{i}$, the 95% sample quantile of the expression level of the *i*th gene, for all genes. $M_{i}$ $M_{i}$ can be considered as a robust estimator of the maximum expression level of the *i*th gene. We defined trimmed robust maximum log-expressions as $L_{i}=\log_{2} {(M}_{i}+1)$, and removed those genes with *L_i_*<3.0, or equivalently, $M_{i}<7.0$.
2. Secondly, to avoid spurious findings due to outliers, we winsorized the data at 1% and 99% levels. Specifically, if an observation was less (greater) than 99% of the data, we replaced its value by the 1% (99%) sample quantile. We also removed genes for which their expression was winsorized in more than half of the samples.
3. Thirdly, we computed the IQR of all genes in each dataset and kept those genes with IQR greater or equal to the median IQR for subsequent analyses.
4. Finally, we selected those genes that had significant (*p*<0.05 without multiple testing adjustment) association with the GRSS, as determined by the Pearson correlation test. Note that we chose Pearson correlation test instead of the Spearman’s rank-based correlation test because: (a) after extensive pre-processing, the empirical distributions of the processed gene expression data are approximately normal with little granularity, and (b) by design, the proposed multilayer statistical learning framework is based on both linear dimensionality reduction and a multivariate linear predictor; therefore, Pearson’s correlation is a more direct measure of marginal linear association than Spearman’s correlation.

Table S6 summarizes number of genes selected for further analyses.

*Batch effect correction*

Because the study spanned three winter seasons (October 2012 through April 2015) samples were processed in six library batches. This resulted in significant batch effects in the total number of mapped reads (Fig S23). In addition, analysis of variance (ANOVA) F-test with false discovery rate (FDR) controlled at 0.05 level, found 3,984 genes (28.8% of the reported transcriptome) had significantly different mean expressions across batches. Based on these observations, we applied ComBat to remove batch effects ^40^. After applying ComBat, none of the genes had significant batch effects based on ANOVA F-test, and pairwise correlation analysis showed that the average Pearson correlation between the original and ComBat processed data was 0.987. This suggests that ComBat only removed batch effects with minimum impact on the remaining information.

*Nasal microbiome genomic DNA extraction*

Total genomic DNA was extracted from the nasal samples using a modification of the ZymoBIOMICS^TM^ DNA Miniprep Kit (Zymo Research, Irvine, CA) and FastPrep mechanical lysis (MPBio, Solon, OH). 16S ribosomal DNA (rRNA) was amplified with Phusion High-Fidelity polymerase (Thermo Scientific, Waltham, MA) and dual indexed primers specific to the V1-V3 (8F: 5’ AGAGTTTGATCCTGGCTCAG 3’; 534R: 3’ ATTACCGCGGCTGCTGG 5’) hypervariable regions ^41^. Amplicons were pooled and paired-end sequenced on an Illumina MiSeq (Illumina, San Diego, CA) in the University of Rochester Genomics Research Center. Positive controls comprised of mock communities and negative controls consisting of sterile saline were processed in parallel to the biological samples were used to validate the sequencing data and verify the absence of contamination, which was actively mitigated by UV irradiation of all reagents and sample processing equipment.

*Nasal microbiome bioinformatic analysis*

Raw data from the Illumina MiSeq was first converted into FASTQ format 2x312 paired end sequence files using the bcl2fastq program, version 1.8.4, provided by Illumina. Format conversion was performed without de-multiplexing and the EAMMS algorithm was disabled. All other settings were default. Reads were multiplexed using a configuration described previously ^41^. Briefly, for both reads in a pair, the first 12 bases were a barcode, which was followed by a primer, then a heterogeneity spacer, and then the target 16S rRNA sequence. QIIME 1.9.1 ^33^ was used to extract the barcodes into a separate file for importing into QIIME 2 V2018.11) ^32^, which was used to perform all subsequent processing. Reads were demultiplexed requiring exact barcode matches, and 16S primers were removed allowing 20% mismatches and requiring at least 18 bases. Cleaning, joining, and denoising were performed using DADA2 ^42^: forward reads were truncated to 275 bps and reverse reads to 260 bps, error profiles were learned with a sample of one million reads, and a maximum expected error of two was allowed. Taxonomic classification was performed with a custom naïve Bayesian classifier trained on the August, 2013 release of GreenGenes ^34^. Sequence variants that could not be classified at least at the phylum level were discarded. Sequencing variants observed fewer than ten times total, or in only one sample, were discarded. Samples with fewer than 11,500 reads were discarded.

Nasal microbiota data were available for a total of 471 operational taxonomic units (OTUs). After an initial filtering to remove those OTUs for which neither genus or species was discernable, or only detected in fewer than three subjects, 148 OTUs remained. We noticed that many of these OTUs were detected in only a handful of subjects. To ensure that statistical modeling is not solely dependent on a small subset of samples, we decided to focus our analyses on the 15 OTUs that were detected in at least 50% of the samples. A full list of these 15 OTUs is provided in Table S7.

*Feature weight calculation*

Here we show how individual features weights were calculated based on our proposed integrative models. In principal component analysis, each PC can be represented by a linear of input features. Specifically, let $x_{s,ij}$ be the value of the $i$th feature in the $s$th type of data of the $j$th subject (e.g., the expression level of the $i$th gene in CD4 for subject $j$) and $PC_{s,kj}$ be the value of the $k$th PC of this dataset for subject $j$. We have

$$PC_{s,kj}=\sum_{i=1}^{p_{s}} {(x}_{s,ij}-\bar{x}_{s,i\cdot})w_{s,ik}=c_{s,k}+\sum_{i=1}^{p_{s}} x_{s,ij}w_{s,ik}, c_{s,k}=-\sum_{i=1}^{p_{s}} \bar{x}_{s,i\cdot}\cdot w_{s,ik}.$$

Here $w_{s,ik}$ is the $ik$th element in the loading matrix, $c_{s,k}$ is a constant resulted from the centering step in PCA. If a secondary PCA was applied to PCs computed from all types of data as in Method 5, we can similarly represent the second stage PCs (denoted by $SPC_{lj}$) as

$$SPC_{lj}=c_{l}+\sum_{s=1}^{S} \sum_{k=1}^{K} PC_{s,kj}u_{s,kl}=\tilde{c}_{l}+\sum_{s=1}^{S} \sum_{k=1}^{K_{s}} \sum_{i=1}^{p_{s}} x_{s,ij}w_{s,ik}u_{s,kl}=\tilde{c}_{l}+\sum_{s=1}^{S} \sum_{i=1}^{p_{s}} x_{s,ij}\tilde{w}_{s,il}.$$

Here $u_{s,k}$ is the loading of $PC_{s,k\cdot}$ associated with $SPC_{l\cdot}$ in the second-stage PCA; $\tilde{w}_{s,il}=\sum_{k=1}^{K_{s}} w_{s,ik}u_{s,kl}$ is the combined loading of $x_{s,i\cdot}$ (the original features) associated with $SPC_{l\cdot}$; $c_{l}$ and $\tilde{c}_{l}$ are constants resulted from the centering steps.

In final integrative models, per-datatype PCs or second-stage PCs were used as covariates in elastic-net regression to predict GRSS. Using Method 5 as an example, the final model can be represented as

$$\begin{aligned} {\hat{\mathrm{GRSS}}}_{j}=\hat{\beta}_{0}+\sum_{l=1}^{L} SPC_{lj}\hat{\beta}_{l}=\hat{\beta}_{0}+\sum_{l=1}^{L} \left( \tilde{c}_{l}+\sum_{s=1}^{S} \sum_{i=1}^{p_{s}} x_{s,ij}\tilde{w}_{s,il} \right)\hat{\beta}_{l}=\tilde{\beta}_{0}+\sum_{s=1}^{S} \sum_{i=1}^{p_{s}} x_{s,ij}\check{w}_{s,i}.\#\left( 2 \right) \end{aligned}$$

Here $\hat{\beta}_{0}$ and $\hat{\beta}_{l}$ are the linear coefficients estimated by glmnet; $\tilde{\beta}_{0}=\hat{\beta}_{0}+\sum_{i=1}^{L} \tilde{c}_{l}\hat{\beta}_{l}$ is a constant; and $\check{w}_{s,i}$ is the final combined weight that quantifies the contribution of $x_{s,i\cdot}$ to ${\hat{\mathrm{GRSS}}}_{\cdot}$, the predicted GRSS. In this study, we used the $\check{w}_{s,i}$ as inputs in transcription factor analyses and to select informative genes for pathway analyses.

Supplementary Tables:

|  | | GRSS | |  |
| --- | --- | --- | --- | --- |
| Variable | Overall, N = 134^1^ | Mild, N = 50^1^ | Severe, N = 84^1^ | p-value^2^ |
| **Age** | 3.08 (2.44) | 3.20 (2.64) | 3.01 (2.33) | 0.683 |
| **continuous GRSS** | 4.51 (2.71) | 1.55 (0.97) | 6.27 (1.67) | <0.001 |
| **Sample.Days.Since.Onset** | 4.79 (2.04) | 4.65 (2.11) | 4.87 (2.00) | 0.557 |
| **Sex** |  |  |  | 0.723 |
| Female | 70 (52%) | 25 (50%) | 45 (54%) |  |
| Male | 64 (48%) | 25 (50%) | 39 (46%) |  |
| **Ethnicity** |  |  |  | 0.461 |
| Hispanic or Latino | 20 (15%) | 9 (18%) | 11 (13%) |  |
| Non-Hispanic or Non-Latino | 114 (85%) | 41 (82%) | 73 (87%) |  |
| **Race** |  |  |  | 0.125 |
| Black.African.American | 29 (22%) | 16 (32%) | 13 (16%) |  |
| Caucasian | 82 (63%) | 28 (56%) | 54 (68%) |  |
| Other | 19 (15%) | 6 (12%) | 13 (16%) |  |
| **RSV.Strain** |  |  |  | 0.466 |
| A | 76 (58%) | 26 (54%) | 50 (61%) |  |
| B | 54 (42%) | 22 (46%) | 32 (39%) |  |
| **Antibiotic.Use.LE.v11** |  |  |  | 0.218 |
| No | 66 (77%) | 17 (89%) | 49 (73%) |  |
| Yes | 20 (23%) | 2 (11%) | 18 (27%) |  |
| ^1^Mean (SD); n (%) | | | | |
| ^2^Welch Two Sample t-test; Fisher's exact test | | | | |

**Table A**: Demographics table for 134 subjects analyzed in this study. Subject level variables are stratified by Global Respiratory Severity Score (GRSS).

|  | | GRSS | |  |
| --- | --- | --- | --- | --- |
| Variable | Overall, N = 61^1^ | Mild, N = 21^1^ | Severe, N = 40^1^ | p-value^2^ |
| **Age** | 3.32 (2.55) | 2.98 (2.42) | 3.50 (2.63) | 0.446 |
| **continuous GRSS** | 4.59 (2.67) | 1.70 (1.14) | 6.11 (1.86) | <0.001 |
| **Sample.Days.Since.Onset** | 4.66 (1.70) | 4.19 (1.54) | 4.90 (1.75) | 0.110 |
| **Sex** |  |  |  | 0.788 |
| Female | 29 (48%) | 9 (43%) | 20 (50%) |  |
| Male | 32 (52%) | 12 (57%) | 20 (50%) |  |
| **Ethnicity** |  |  |  | 0.405 |
| Hispanic or Latino | 6 (9.8%) | 3 (14%) | 3 (7.5%) |  |
| Non-Hispanic or Non-Latino | 55 (90%) | 18 (86%) | 37 (92%) |  |
| **Race** |  |  |  | 0.359 |
| Black.African.American | 13 (22%) | 7 (33%) | 6 (16%) |  |
| Caucasian | 36 (62%) | 11 (52%) | 25 (68%) |  |
| Other | 9 (16%) | 3 (14%) | 6 (16%) |  |
| **RSV.Strain** |  |  |  | >0.999 |
| A | 29 (48%) | 10 (48%) | 19 (49%) |  |
| B | 31 (52%) | 11 (52%) | 20 (51%) |  |
| **Antibiotic.Use.LE.v11** |  |  |  | >0.999 |
| No | 37 (88%) | 9 (90%) | 28 (88%) |  |
| Yes | 5 (12%) | 1 (10%) | 4 (12%) |  |
| ^1^Mean (SD); n (%) | | | | |
| ^2^Welch Two Sample t-test; Fisher's exact test | | | | |

**Table B**: Demographics table for 61 subjects analyzed in the nasal transcriptome, CD4 transcriptome, nasal microbiome integration model. Subject level variables are stratified by Global Respiratory Severity Score (GRSS).

|  | | GRSS | |  |
| --- | --- | --- | --- | --- |
| Variable | Overall, N = 35^1^ | Mild, N = 15^1^ | Severe, N = 20^1^ | p-value^2^ |
| **Age** | 3.15 (2.40) | 3.69 (2.43) | 2.76 (2.36) | 0.265 |
| **continuous GRSS** | 4.43 (2.93) | 1.70 (0.90) | 6.47 (2.12) | <0.001 |
| **Sample.Days.Since.Onset** | 4.54 (1.62) | 4.60 (1.76) | 4.50 (1.54) | 0.862 |
| **Sex** |  |  |  | 0.097 |
| Female | 20 (57%) | 6 (40%) | 14 (70%) |  |
| Male | 15 (43%) | 9 (60%) | 6 (30%) |  |
| **Ethnicity** |  |  |  | >0.999 |
| Hispanic or Latino | 4 (11%) | 2 (13%) | 2 (10%) |  |
| Non-Hispanic or Non-Latino | 31 (89%) | 13 (87%) | 18 (90%) |  |
| **Race** |  |  |  | 0.431 |
| Black.African.American | 8 (24%) | 5 (33%) | 3 (17%) |  |
| Caucasian | 20 (61%) | 9 (60%) | 11 (61%) |  |
| Other | 5 (15%) | 1 (6.7%) | 4 (22%) |  |
| **RSV.Strain** |  |  |  | 0.321 |
| A | 20 (57%) | 7 (47%) | 13 (65%) |  |
| B | 15 (43%) | 8 (53%) | 7 (35%) |  |
| **Antibiotic.Use.LE.v11** |  |  |  | 0.530 |
| No | 16 (84%) | 3 (75%) | 13 (87%) |  |
| Yes | 3 (16%) | 1 (25%) | 2 (13%) |  |
| ^1^Mean (SD); n (%) | | | | |
| ^2^Welch Two Sample t-test; Fisher's exact test | | | | |

**Table C**: Demographics table for 35 subjects analyzed in the CD4, CD8, and CD19 transcriptome integration model. Subject level variables are stratified by Global Respiratory Severity Score (GRSS).

| Integration Method | Cumulative proportion of variance threshold | Reduced features used  in elastic net model | Variable selection method | # of features selected | CV RSS | CV Correlation |
| --- | --- | --- | --- | --- | --- | --- |
| Method 1 | 0.7 | 14 NT PCs / 23 CD4 PCs / 15 OTUs | 1se | 12 | 2.46 | 0.82 |
| Method 1 | 0.7 | 14 NT PCs / 23 CD4 PCs / 15 OTUs | min | 28 | 4.02 | 0.79 |
| **Method 1** | **0.8** | **27 NT PCs / 35 CD4 PCs / 15 OTUs** | **1se** | **16** | **1.64** | **0.88** |
| Method 1 | 0.8 | 27 NT PCs / 35 CD4 PCs / 15 OTUs | min | 31 | 2.47 | 0.85 |
| Method 1 | 0.9 | 50 NT PCs / 53 CD4 PCs / 15 OTUs | 1se | 7 | 2.69 | 0.80 |
| Method 1 | 0.9 | 50 NT PCs / 53 CD4 PCs / 15 OTUs | min | 15 | 1.80 | 0.87 |
| Method 2 | 0.7 | 17 combined PCs | 1se | 2 | 4.38 | 0.63 |
| Method 2 | 0.7 | 17 combined PCs | min | 6 | 7.59 | 0.61 |
| Method 2 | 0.8 | 26 combined PCs | 1se | 2 | 4.38 | 0.63 |
| Method 2 | 0.8 | 26 combined PCs | min | 6 | 7.59 | 0.61 |
| Method 2 | 0.9 | 39 combined PCs | 1se | 2 | 4.38 | 0.63 |
| Method 2 | 0.9 | 39 combined PCs | min | 6 | 7.59 | 0.61 |
| Method 3 | 0.7 | 14 NT PCs / 23 CD4 PCs / 15 OTUs  16 combined PCs | 1se | 8 | 4.06 | 0.68 |
| Method 3 | 0.7 | 14 NT PCs / 23 CD4 PCs / 15 OTUs  16 combined PCs | min | 12 | 6.48 | 0.60 |
| Method 3 | 0.8 | 27 NT PCs / 35 CD4 PCs / 15 OTUs  27 combined PCs | 1se | 16 | 3.58 | 0.74 |
| Method 3 | 0.8 | 27 NT PCs / 35 CD4 PCs / 15 OTUs  27 combined PCs | min | 19 | 4.18 | 0.71 |
| Method 3 | 0.9 | 50 NT PCs / 53 CD4 PCs / 15 OTUs  42 combined PCs | 1se | 0 | NA | NA |
| Method 3 | 0.9 | 50 NT PCs / 53 CD4 PCs / 15 OTUs  42 combined PCs | min | 6 | 4.16 | 0.65 |
| Method 4 | 0.7 | 14 NT PCs / 23 CD4 PCs / 6 OTU PCs | 1se | 17 | 2.72 | 0.83 |
| Method 4 | 0.7 | 14 NT PCs / 23 CD4 PCs / 6 OTU PCs | min | 23 | 4.61 | 0.77 |
| Method 4 | 0.8 | 27 NT PCs / 35 CD4 PCs / 8 OTU PCs | 1se | 9 | 1.96 | 0.85 |
| Method 4 | 0.8 | 27 NT PCs / 35 CD4 PCs / 8 OTU PCs | min | 35 | 3.45 | 0.80 |
| Method 4 | 0.9 | 50 NT PCs / 53 CD4 PCs / 10 OTU PCs | 1se | 8 | 2.07 | 0.84 |
| Method 4 | 0.9 | 50 NT PCs / 53 CD4 PCs / 10 OTU PCs | min | 14 | 1.83 | 0.87 |
| Method 5 | 0.7 | 14 NT PCs / 23 CD4 PCs / 6 OTU PCs  16 combined PCs | 1se | 0 | NA | NA |
| Method 5 | 0.7 | 14 NT PCs / 23 CD4 PCs / 6 OTU PCs  16 combined PCs | min | 12 | 7.39 | 0.58 |
| Method 5 | 0.8 | 27 NT PCs / 35 CD4 PCs / 8 OTU PCs  27 combined PCs | 1se | 7 | 3.54 | 0.72 |
| Method 5 | 0.8 | 27 NT PCs / 35 CD4 PCs / 8 OTU PCs  27 combined PCs | min | 23 | 6.11 | 0.65 |
| Method 5 | 0.9 | 50 NT PCs / 53 CD4 PCs / 10 OTU PCs  42 combined PCs | 1se | 5 | 4.44 | 0.63 |
| Method 5 | 0.9 | 50 NT PCs / 53 CD4 PCs / 10 OTU PCs  42 combined PCs | min | 8 | 3.89 | 0.69 |

**Table D:** Performance for integration methods on CD4 / NT / OTU data integration. Column 1 shows the integration method from Fig 1/ Column 2 shows the cumulative proportion of variance threshold used to select the number of PCs to include. Column 3 shows the number of PCs, combined PCS, and/or OTUs input into the elastic net model. Column 4 shows the variable selection methods used by the elastic net model, either the value of 𝜆 that gives the minimum mean cross-validated error (min) or the value of 𝜆 that gives the most regularized model such that the cross-validated error is within one standard error of the minimum (1se). Column 5 shows the number of features selected by the elastic net model. Columns 6 & 7 show performance metrics for the elastic net model: the residual sum of squares (RSS) and correlation for GRSS based on 10-fold cross-validation (CV).

|  | Total number of samples | Samples that passed QC and were used for subsequent analyses |
| --- | --- | --- |
| NT | 119 | 106 |
| CD4 | 98 | 92 |
| CD8 | 52 | 51 |
| CD19 | 68 | 66 |

**Table E**: Summary of the quality assurance analysis.

|  | Number of genes after filtering based on 95% quantile | Number of genes after filtering based on IQR | Pearson correlation with GRSS (p value<0.05) |
| --- | --- | --- | --- |
| NT | 13819 | 6844 | 993 |
| CD4 | 11756 | 5812 | 454 |
| CD8 | 11786 | 5827 | 333 |
| CD19 | 12177 | 6026 | 662 |

**Table F**: Number of genes selected for integrative analyses.

| **Phylum** | **Class** | **Order** | **Family** | **Genus** | **Species** |
| --- | --- | --- | --- | --- | --- |
| Actinobacteria | Actinobacteria | Actinomycetales | Actinomycetaceae | Actinomyces | - |
| Actinobacteria | Actinobacteria | Actinomycetales | Micrococcaceae | Rothia | Mucilaginosa |
| Actinobacteria | Actinobacteria | Actinomycetales | Propionibacteriaceae | Propionibacterium | Acnes |
| Actinobacteria | Actinobacteria | Corynebacteriales | Corynebacteriaceae | Corynebacterium | Unknown |
| Bacteroidetes | Bacteroidia | Bacteroidales | Bacteroidaceae | Prevotella | Melaninogenica |
| Firmicutes | Bacilli | Bacillales | Staphylococcaceae | Staphylococcus | Epidermidis |
| Firmicutes | Bacilli | Bacillales | Staphylococcaceae | Staphylococcus | Unknown |
| Firmicutes | Bacilli | Lactobacillales | Carnobacteriaceae | Alloiococcus | - |
| Firmicutes | Bacilli | Lactobacillales | Streptococcaceae | Streptococcus | Unknown |
| Firmicutes | Negativicutes | Vellionellales | Veillonellaceae | Veillonella | Dispar |
| Firmicutes | Negativicutes | Vellionellales | Veillonellaceae | Veillonella | Unknown |
| Proteobacteria | Gammaproteobacteria | Pseudomonadales | Moraxellaceae | Moraxella | Unknown |
| Proteobacteria | Gammaproteobacteria | Pseudomonadales | Pseudomonadaceae | Pseudomonas | Unknown |
| Proteobacteria | Gammaproteobacteria | Pasteurellales | Pasteurellaceae | Haemophilus | Influenzae |
| Proteobacteria | Gammaproteobacteria | Pasteurellales | Pasteurellaceae | Haemophilus | Unknown |

**Table G:** List of all 15 OTUs in nasal microbiota used in integrative analysis. “-” in the last column represents all species in that genus, “Unknown” in the last column indicates that we did not capture sufficient sequence variation to discriminate between closely related taxa at the species level.
