# Supplementary figures and images for: A systems genomics approach uncovers molecular associates of RSV severity
Source: PLoS Comput Biol. 2021 Dec 28;17(12):e1009617. doi: 10.1371/journal.pcbi.1009617 (PMC8746750; doi:10.1371/journal.pcbi.1009617)

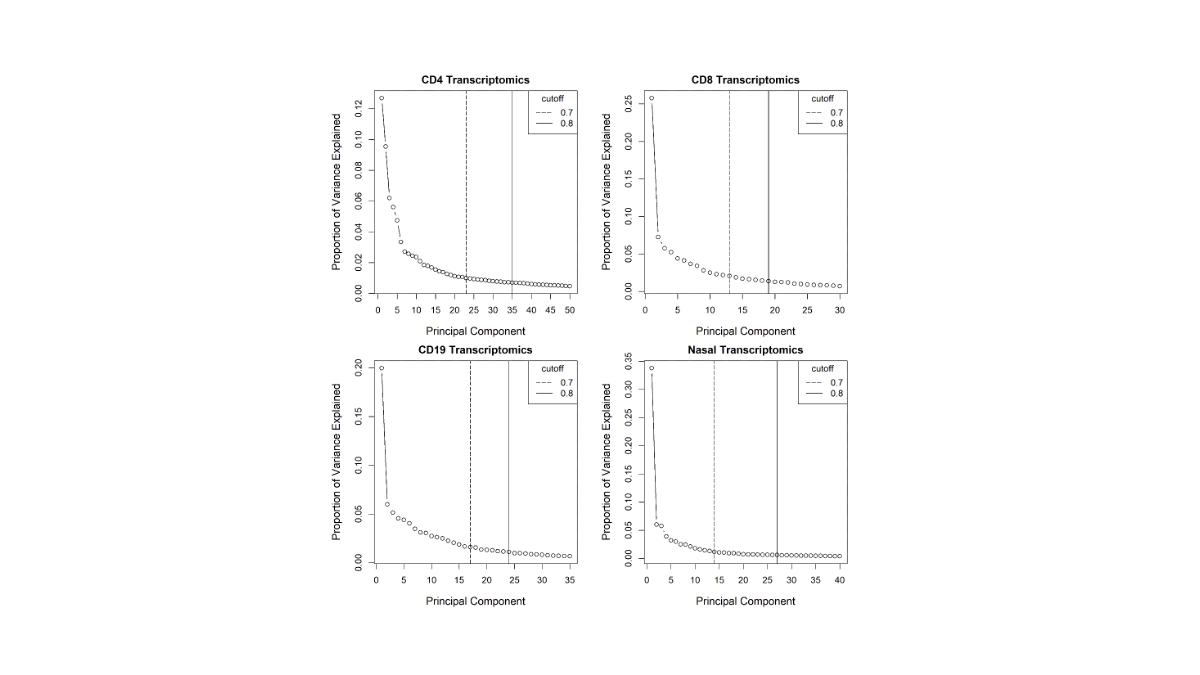

Supplement: S1 Fig — Vertical lines denote the number of principal components that explain 70% of the variation (dashed) or 80% of the variation (solid). (TIFF) [file pcbi.1009617.s002.tiff]

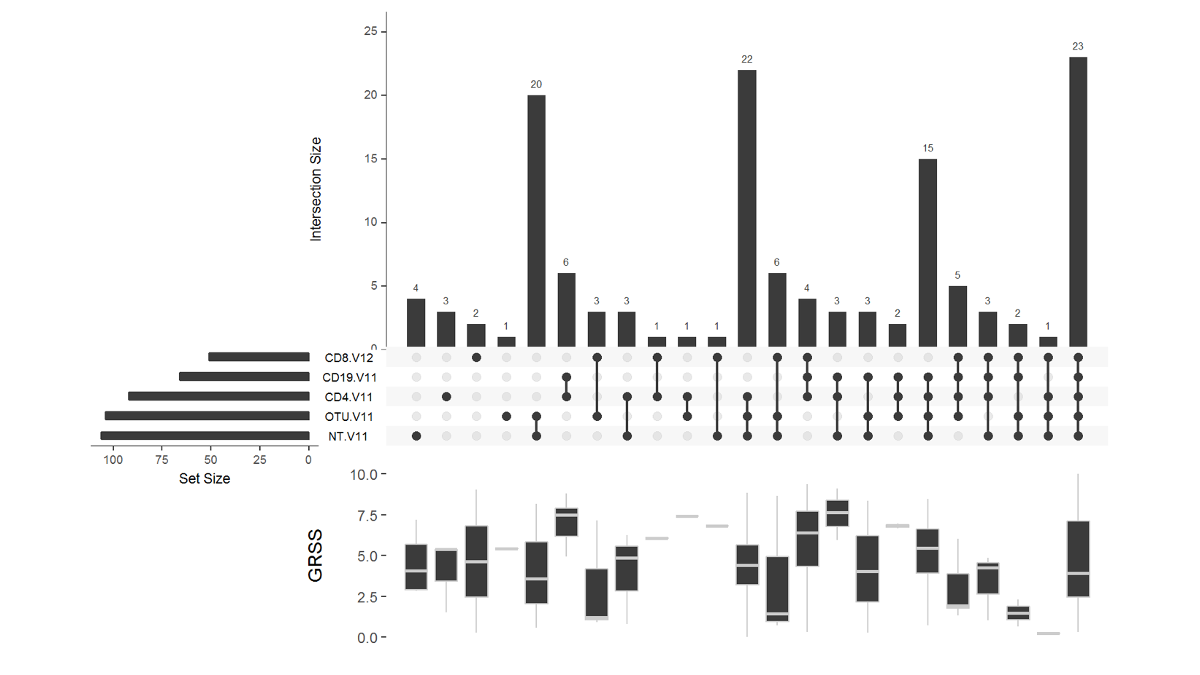

Supplement: S2 Fig — (TIFF) [file pcbi.1009617.s003.tiff]

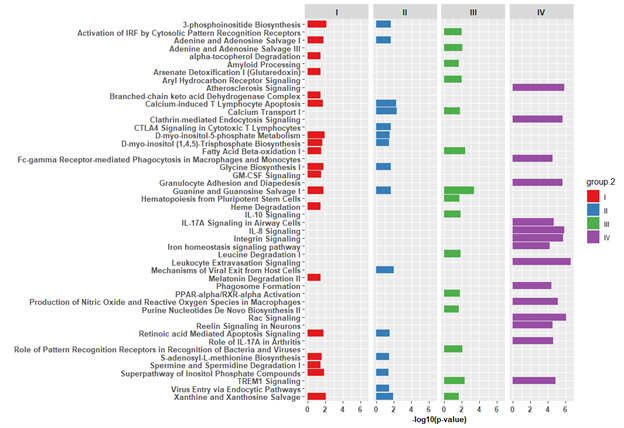

Supplement: S3 Fig — Canonical pathways defined by the subset of top 200 (I), 1st quartile (II), 2nd quartile (III) and the set of 993 genes (IV) genes with expression associated with clinical severity were identified using Ingenuity Pathway Analysis (IPA). Shown are the 15 pathways with the lowest significant p-values using Fisher’s exact test. (TIFF) [file pcbi.1009617.s004.tiff]

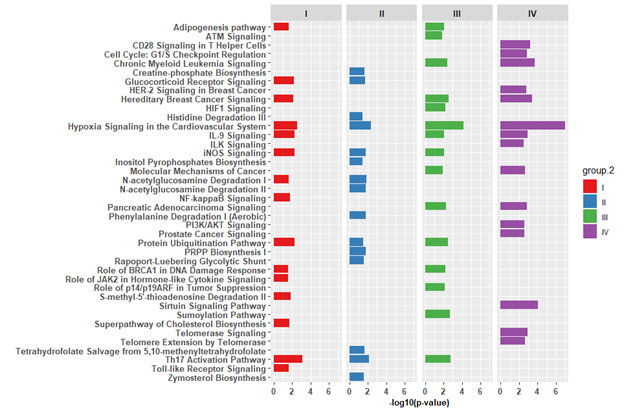

Supplement: S4 Fig — Canonical pathways defined by the subset of top 200 (I), 1st quartile (II), 2nd quartile (III) and the set of 454 genes (IV) genes with expression associated with clinical severity were identified using Ingenuity Pathway Analysis (IPA). Shown are the 15 pathways with the lowest significant p-values using Fisher’s exact test. (TIFF) [file pcbi.1009617.s005.tiff]

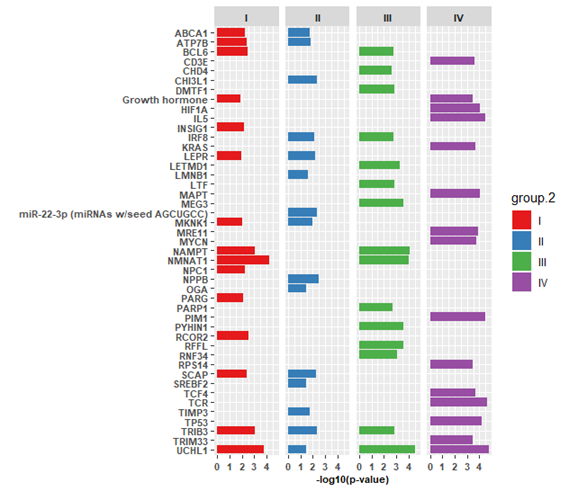

Supplement: S5 Fig — Upstream regulators defined by the subset of top 200 (I), 1st quartile (II), 2nd quartile (III) and the set of 993 genes (IV) genes with expression associated with clinical severity were identified using Ingenuity Pathway Analysis (IPA). Shown are the 15 regulators with the lowest significant p-values using Fisher’s exact test. (TIFF) [file pcbi.1009617.s006.tiff]

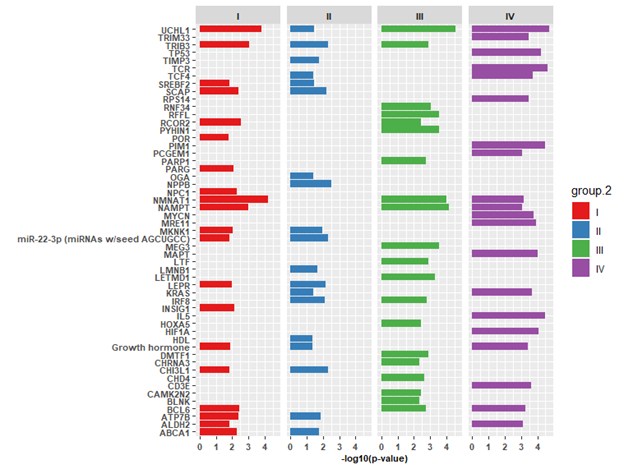

Supplement: S6 Fig — Upstream regulators defined by the subset of top 200 (I), 1st quartile (II), 2nd quartile (III) and the set of 454 genes (IV) genes with expression associated with clinical severity were identified using Ingenuity Pathway Analysis (IPA). Shown are the 15 regulators with the lowest significant p-values. (TIFF) [file pcbi.1009617.s007.tiff]

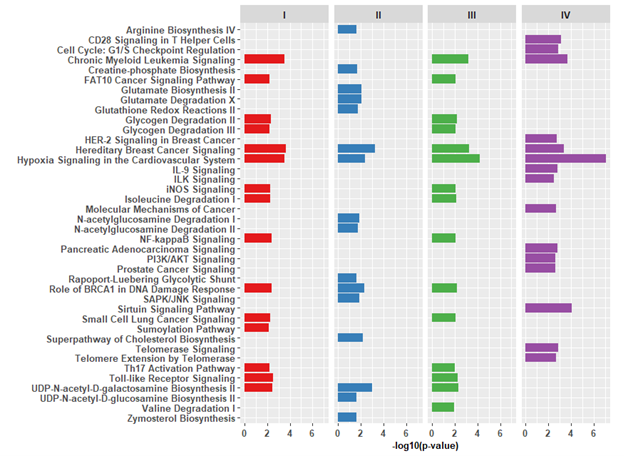

Supplement: S7 Fig — Canonical pathways defined by the subset of top 200 (I), 1st quartile (II), 2nd quartile (III) and the set of 454 genes (IV) genes with expression associated with clinical severity were identified using Ingenuity Pathway Analysis (IPA). Shown are the 15 pathways with the lowest significant p-values using Fisher’s exact test. (TIFF) [file pcbi.1009617.s008.tiff]

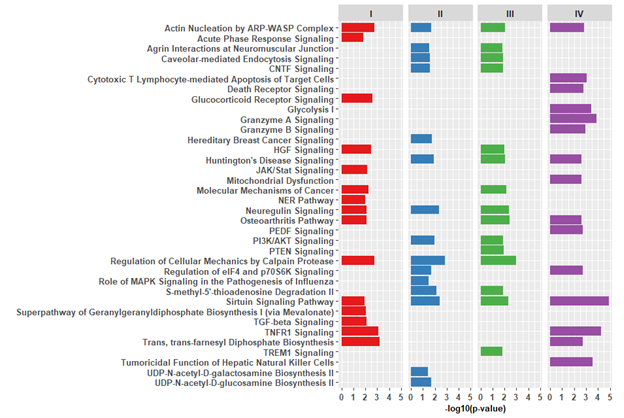

Supplement: S8 Fig — Canonical pathways defined by the subset of top 200 (I), 1st quartile (II), 2nd quartile (III) and the set of 333 genes (IV) genes with expression associated with clinical severity were identified using Ingenuity Pathway Analysis (IPA). Shown are the 15 pathways with the lowest significant p-values using Fisher’s exact test. (TIFF) [file pcbi.1009617.s009.tiff]

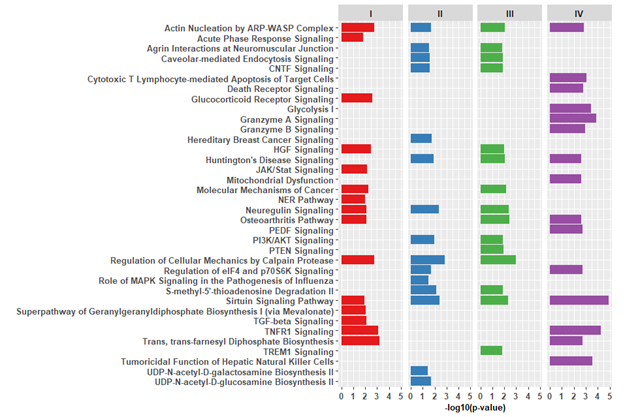

Supplement: S9 Fig — Canonical pathways defined by the subset of top 200 (I), 1st quartile (II), 2nd quartile (III) and the set of 662 genes (IV) genes with expression associated with clinical severity were identified using Ingenuity Pathway Analysis (IPA). Shown are the 15 pathways with the lowest significant p-values using Fisher’s exact test. (TIFF) [file pcbi.1009617.s010.tiff]

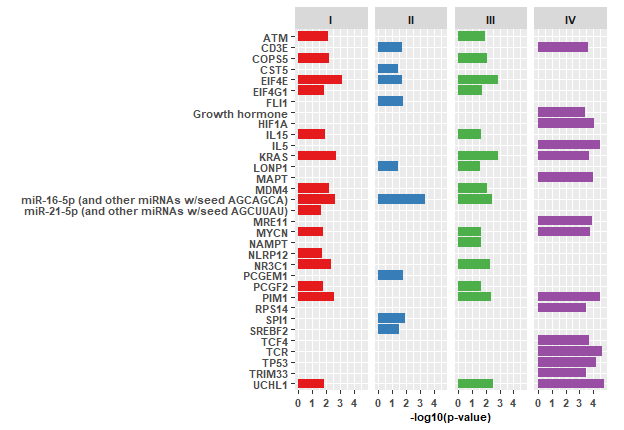

Supplement: S10 Fig — Upstream regulators defined by the subset of top 200 (I), 1st quartile (II), 2nd quartile (III) and the set of 454 genes (IV) genes with expression associated with clinical severity were identified using Ingenuity Pathway Analysis (IPA). Shown are the 15 regulators with the lowest significant p-values. (TIFF) [file pcbi.1009617.s011.tiff]

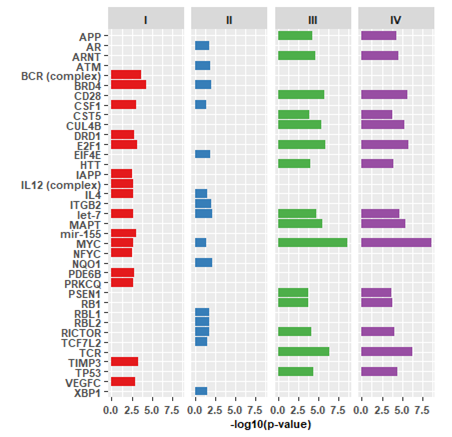

Supplement: S11 Fig — Upstream regulators defined by the subset of top 200 (I), 1st quartile (II), 2nd quartile (III) and the set of 333 genes (IV) genes with expression associated with clinical severity were identified using Ingenuity Pathway Analysis (IPA). Shown are the 15 regulators with the lowest significant p-values. (TIFF) [file pcbi.1009617.s012.tiff]

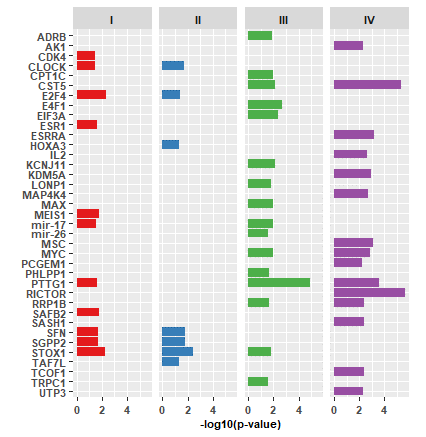

Supplement: S12 Fig — Upstream regulators defined by the subset of top 200 (I), 1st quartile (II), 2nd quartile (III) and the set of 662 genes (IV) genes with expression associated with clinical severity were identified using Ingenuity Pathway Analysis (IPA). Shown are the 15 regulators with the lowest significant p-values. (TIFF) [file pcbi.1009617.s013.tiff]

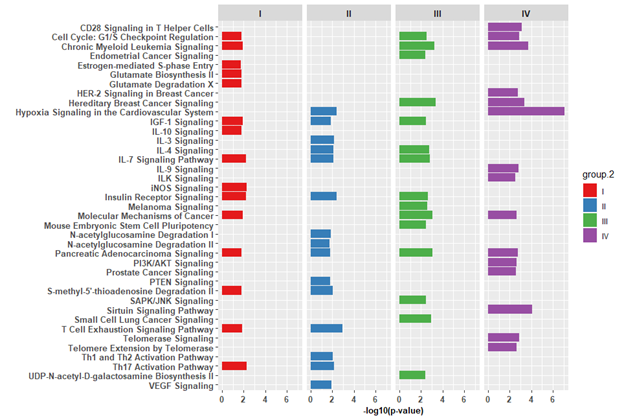

Supplement: S13 Fig — Canonical pathways defined by the subset of top 200 (I), 1st quartile (II), 2nd quartile (III) and the set of 454 genes (IV) genes with expression associated with clinical severity were identified using Ingenuity Pathway Analysis (IPA). Shown are the 15 pathways with the lowest significant p-values using Fisher’s exact test. (TIFF) [file pcbi.1009617.s014.tiff]

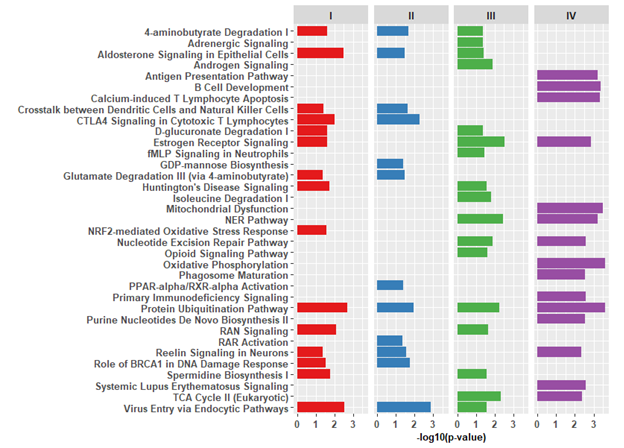

Supplement: S14 Fig — Canonical pathways defined by the subset of top 200 (I), 1st quartile (II), 2nd quartile (III) and the set of 662 genes (IV) genes with expression associated with clinical severity were identified using Ingenuity Pathway Analysis (IPA). Shown are the 15 pathways with the lowest significant p-values using Fisher’s exact test. (TIFF) [file pcbi.1009617.s015.tiff]

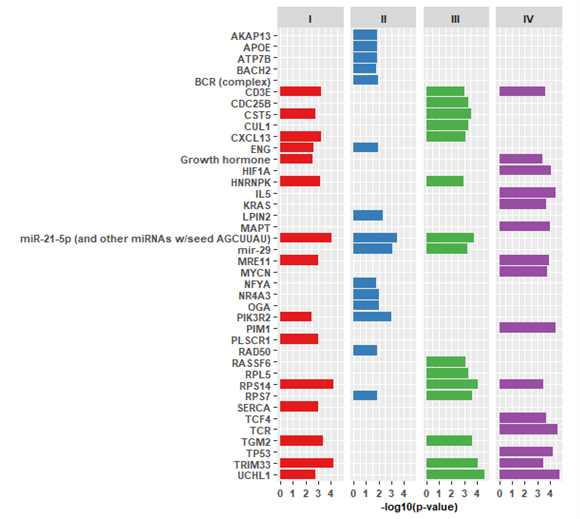

Supplement: S15 Fig — Upstream regulators defined by the subset of top 200 (I), 1st quartile (II), 2nd quartile (III) and the set of 454 genes (IV) genes with expression associated with clinical severity were identified using Ingenuity Pathway Analysis (IPA). Shown are the 15 regulators with the lowest significant p-values. (TIFF) [file pcbi.1009617.s016.tiff]

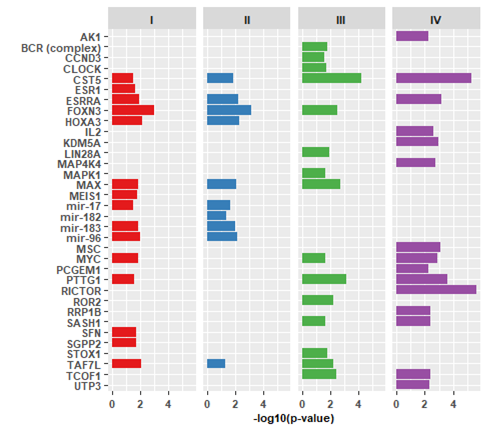

Supplement: S16 Fig — Upstream regulators defined by the subset of top 200 (I), 1st quartile (II), 2nd quartile (III) and the set of 662 genes (IV) genes with expression associated with clinical severity were identified using Ingenuity Pathway Analysis (IPA). Shown are the 15 regulators with the lowest significant p-values. (TIFF) [file pcbi.1009617.s017.tiff]

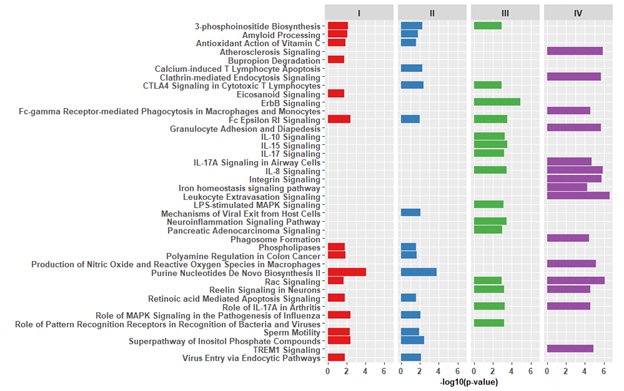

Supplement: S17 Fig — Canonical pathways defined by the subset of top 200 (I), 1st quartile (II), 2nd quartile (III) and the set of 993 genes (IV) genes with expression associated with clinical severity were identified using Ingenuity Pathway Analysis (IPA). Shown are the 15 pathways with the lowest significant p-values using Fisher’s exact test. (TIFF) [file pcbi.1009617.s018.tiff]

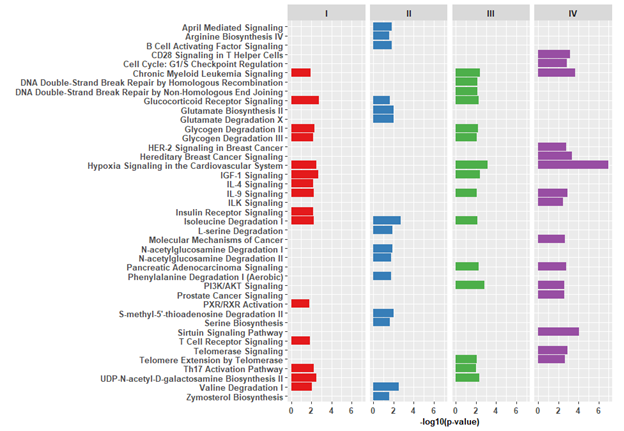

Supplement: S18 Fig — Canonical pathways defined by the subset of top 200 (I), 1st quartile (II), 2nd quartile (III) and the set of 454 genes (IV) genes with expression associated with clinical severity were identified using Ingenuity Pathway Analysis (IPA). Shown are the 15 pathways with the lowest significant p-values using Fisher’s exact test. (TIFF) [file pcbi.1009617.s019.tiff]

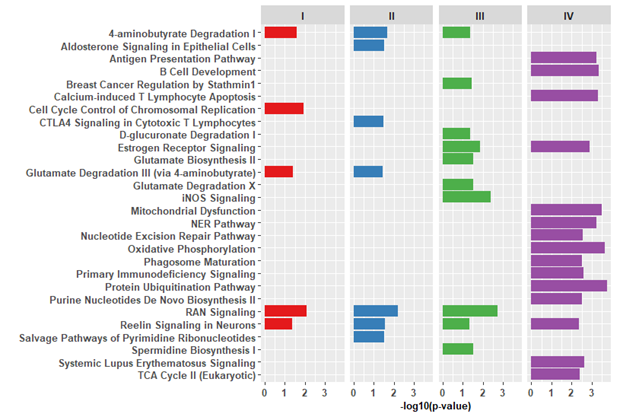

Supplement: S19 Fig — Canonical pathways defined by the subset of top 200 (I), 1st quartile (II), 2nd quartile (III) and the set of 662 genes (IV) genes with expression associated with clinical severity were identified using Ingenuity Pathway Analysis (IPA). Shown are the 15 pathways with the lowest significant p-values using Fisher’s exact test. (TIFF) [file pcbi.1009617.s020.tiff]

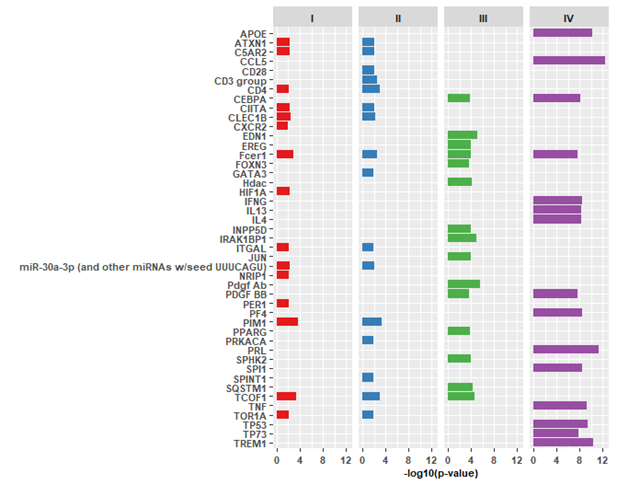

Supplement: S20 Fig — Upstream regulators defined by the subset of top 200 (I), 1st quartile (II), 2nd quartile (III) and the set of 993 genes (IV) genes with expression associated with clinical severity were identified using Ingenuity Pathway Analysis (IPA). Shown are the 15 regulators with the lowest significant p-values using Fisher’s exact test. (TIFF) [file pcbi.1009617.s021.tiff]

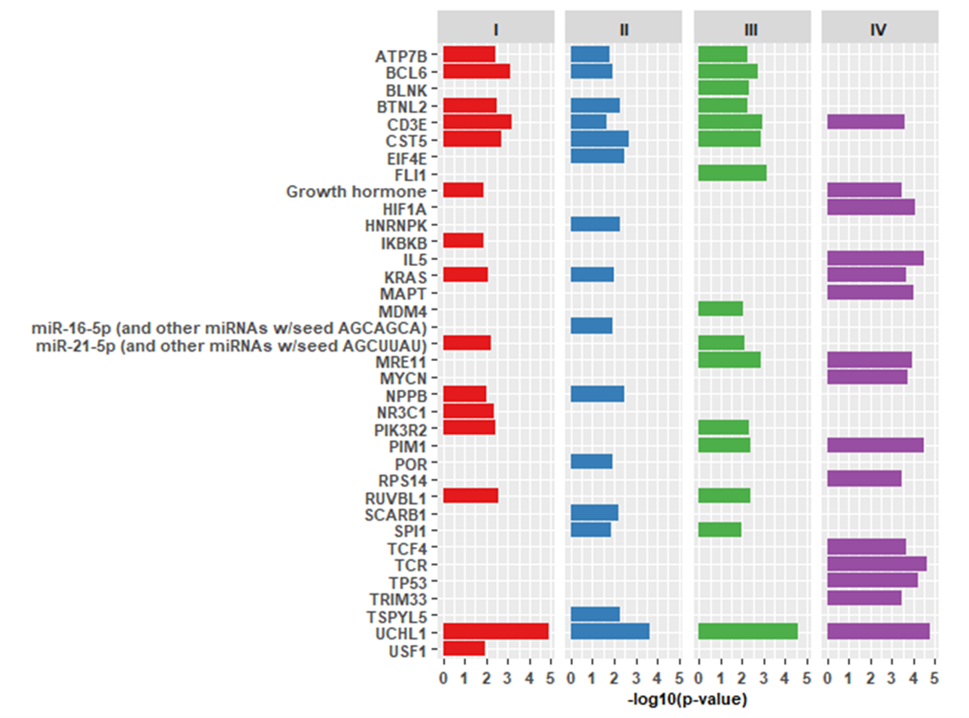

Supplement: S21 Fig — Upstream regulators defined by the subset of top 200 (I), 1st quartile (II), 2nd quartile (III) and the set of 454 genes (IV) genes with expression associated with clinical severity were identified using Ingenuity Pathway Analysis (IPA). Shown are the 15 regulators with the lowest significant p-values. (TIFF) [file pcbi.1009617.s022.tiff]

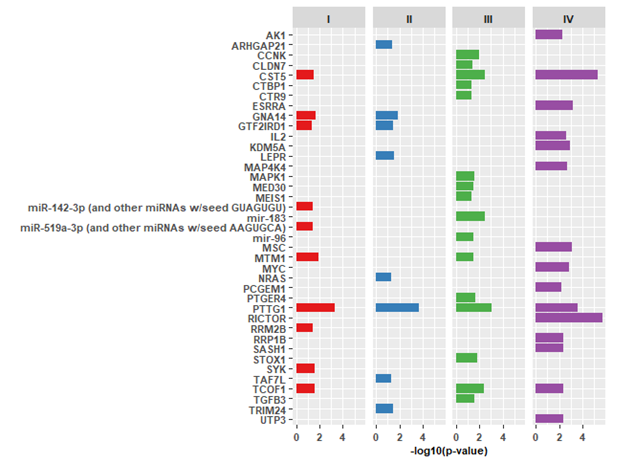

Supplement: S22 Fig — Upstream regulators defined by the subset of top 200 (I), 1st quartile (II), 2nd quartile (III) and the set of 662 genes (IV) genes with expression associated with clinical severity were identified using Ingenuity Pathway Analysis (IPA). Shown are the 15 regulators with the lowest significant p-values. (TIFF) [file pcbi.1009617.s023.tiff]

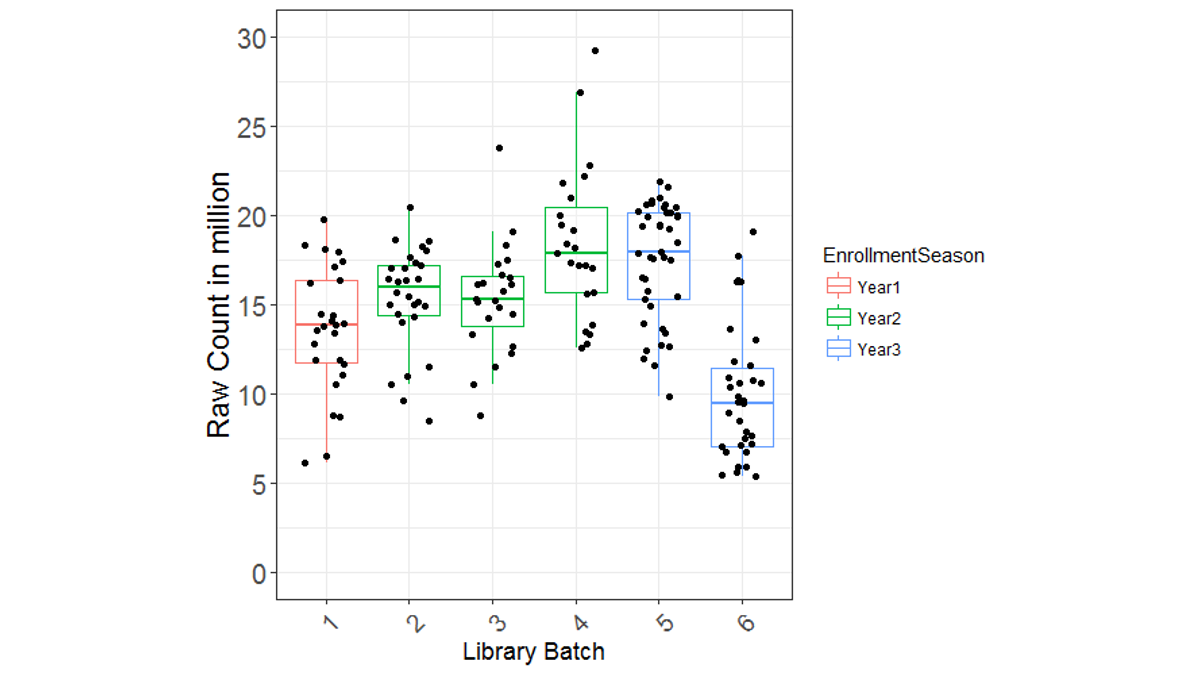

Supplement: S23 Fig — The total number of mapped reads (in millions) is shown on the y-axis stratified by the library preparation batch number; colored boxes denote the enrollment year corresponding to each batch. (TIFF) [file pcbi.1009617.s024.tiff]
