# Supplementary material for: A systems genomics approach uncovers molecular associates of RSV severity
Source: PLoS Comput Biol. 2021 Dec 28;17(12):e1009617. doi: 10.1371/journal.pcbi.1009617 (PMC8746750; doi:10.1371/journal.pcbi.1009617)
Supplement: Attachment — Submitted filename: Response to Reviewers.docx [file pcbi.1009617.s025.docx]

We thank the reviewers for their thoughtful comments. We have incorporated these suggestions to produce a greatly improved manuscript. Below you will find point-by-point responses. The reviewer comments are in italic.

**Reviewer #1:**

*1. METHODS: RNA PROCESSING: Pearson correlation testing of gene expression and GRSS: please specify what kind of normalization and transformation methods are used for correlation. RNA-seq data is not normally distributed.*

**1a:** Due to page limit, the detailed description of the RNA-seq data processing, which includes alignment, normalization, quality assurance based on total mapped reads and between-sample correlation, non-specific filtering based on both maximum gene expression and IQR, and batch effect correction, is provided in Supplementary Text, pages 1-2. For your convenience, we copy the text most relevant to your comment below:

RNA-seq data (CD4, CD8, CD19, and NT) were normalized by Fragments Per Kilobase of transcript per Million mapped reads (FPKM). We applied non-specific filtering based on both maximum expression values and inter-quartile range (IQR) to identify subsets of genes for further investigation.

1. First, we computed $M_{i}$, the 95% sample quantile of the expression level of the *i*th gene, for all genes. $M_{i}$ $M_{i}$ can be considered as a robust estimator of the maximum expression level of the *i*th gene. We defined trimmed robust maximum log-expressions as $L_{i}=\log_{2} {(M}_{i}+1)$, and removed those genes with *L_i_*<3.0, or equivalently, $M_{i}<7.0$.
2. Secondly, to avoid spurious findings due to outliers, we winsorized the data at 1% and 99% levels. Specifically, if an observation was less (greater) than 99% of the data, we replaced its value by the 1% (99%) sample quantile. We also removed genes for which their expression was winsorized in more than half of the samples.
3. Thirdly, we computed the IQR of all genes in each dataset and kept those genes with IQR greater or equal to the median IQR for subsequent analyses.
4. Finally, we selected those genes that had significant (*p*<0.05 without multiple testing adjustment) association with the GRSS, as determined by the Pearson correlation test.

**1b:** “RNA-seq data is not normally distributed”. This is a great question, and we believe it deserves a detailed answer.

First, although raw RNA-seq reads are discrete random variables that are generally modeled by non-normal distributions such as the negative binomial distribution, after non-specific filtering to remove genes with very low reads and outliers with extremely large values, the granularity of the processed RNA-seq data is greatly reduced and their distribution can be considered *approximately* normal. In fact, some recent studies conducted by our group and others [1-5] showed that differential expression analysis tools designed for continuous data can achieve comparable, sometimes even slightly better, performance than those based on discrete models. To better illustrate this point, below are the histograms of four *processed* gene expression data sets, computed from all genes and all subjects:

As we can see from these figures, while these empirical distributions of expression levels are a bit skewed, they are still largely unimodal and perhaps more importantly, “smooth” (e.g., with very little granularity that are typical for discrete distributions such as negative binomial distribution). You may have noticed that, even after outlier detection and Winsorization, there are still some visible “outliers” in the far-right regions of these figures. A detailed analysis showed that the right “bump” accounts for only 0.3% of entries, and is largely driven by a few genes with extremely high baseline expression. This is because our outlier detection algorithm was applied to individual genes, so a large value is not necessarily an outlier if it comes from a gene with a naturally high expression level. Take CD4 as an example, the “bump” is largely contributed by these three genes: CD3E (a member of the T-cell receptor-CD3 complex), LDHA (lactate dehydrogenase A), and TRBC2 (T cell receptor beta constant 2). Histograms of these genes are provided in the following figure:

As you can see, all three genes have very high expression compared with other genes, yet the “shape” of these individual histograms are still approximately normal in our opinion.

Secondly, we chose the Pearson correlation test as a marginal feature screening tool prior to model development in part because our final goal is to train a sparse multivariate *linear* predictor of the disease severity (GRSS), and the Pearson correlation test may be more relevant than Spearman’s rank-based correlation test for this purpose. These discussions are now added to Supplementary Text, page 1, section “*Non-specific filtering, winsorization, and univariate screening*”.

Lastly, while we acknowledge that some level of non-normality in the distribution of gene expression and nonlinear relationship between gene expression levels and the GRSS is inevitable, it is our believe that the relationship between gene expression and GRSS is *approximately* linear, which is manifested in the fact that our trained models had good cross-validated prediction accuracy. Furthermore, using both linear dimensionality reduction (PCA) and linear predictive modeling, we were able to derive a multilayer weight backpropagation algorithm that enabled us to perform pathway analyses and thus obtain biological interpretability of the trained models, which in our opinion is a major advantage of our proposed method compared with a nonlinear machine learning approach (e.g., deep neural network). This point is included in the Discussion.

*2. METHODS: PATHWAY ANALYSIS: Combined feature weights used for pathway analysis were compared to the results without those weights – where is this data?*

Biological pathway and regulator results are shown in supplemental figures S3-S18. In these figures, the subset of the top 200 (I), 1st quartile (II), 2nd quartile (III) and the set of all genes (IV) genes with expression associated with clinical severity were selected based on absolute weight value. The 4^th^ group represents an analysis that ignores the feature weights.

*3. RESULTS: 139 infants were enrolled, but supplemental figure 2 represents only 134 subjects.*

Five subjects were removed from the final data used for analysis due to either a lack of transcriptomic data or poor transcriptomic data quality prior to any analysis. We have clarified this apparent discrepancy in the manuscript.

*4. RESULTS: Table S1. Subsets of CD4, CD8 and CD19 were sorted from PBMC, what is the range of cell numbers for each population? Why only some lymphocyte RNA-seq data was available for certain infants? Is this because low cell number count for some patients?*

Data on variation in sorted lymphocyte recovery using this protocol can be found in our prior “methods” publication [6] As can be seen in the data from this publication, the variation in recovery of individual lymphocyte populations, sometimes (though rarely) does not provide sufficient RNA for transcriptome-wide analysis. Additionally, in rare cases, insufficient blood volumes are recovered that preclude attempts to sort individual populations.

*5. RESULTS: Figure 2. It would be nice to explain different colors in figure legend.*

This is a good suggestion. We now added the following text to the caption of Figure 2: “Here light purple circles represent nasal gene expression profiles (NT), light blue ones represent CD4 gene expressions, and gold ones represent nasal microbiome abundance data (OTU). Dark blue ones are principle components (PCs) computed from the original features. Large white circle represents the GRSS, which is the clinical variable of interest of this study.”

*6. RESULTS: Do you have flow cytometry data of the nasal epithelial cells to show similar cell number/type for each subject? Just to make sure the gene expression difference is not due to the difference in cells present in different cell population.*

Cell recovery from nasal brushings does not provide sufficient cell numbers to complete both cytometry and transcriptome-wide analysis. However, we thoroughly interrogated gene expression profiles from nasal samples recovered from asymptomatic infants, as described in our publication of this method/protocol [7], showing that epithelial cell gene expression signatures are predominant. However, transcripts often attributed to other cell types (e.g., immune cells) can be detected usually at low abundance. Given the significant increase in gene expression for some immune cell markers (e.g., T cells, macrophage) in nasal gene expression profiles from severely-affected RSV-infected infants, we have suggested that this is reflective of excessive inflammation at the site of infection/sample collection. This is supported by our published analyses indicating that gene expression significantly increased in severe subjects has both lymphocytic and monocytic signatures [8]

*7. RESULTS: INTEGRATED METHOD DEVELOPMENT: Supplementary Tables 2&3 don’t actually show the comparison between the PCA/elastic net model and other methods for GRSS prediction? Where is the prediction accuracy of method 1 and other methods?*

We apologize for this oversight. Supplemental Table 4 now shows the performance of all 5 methods with different variable selection procedures and thresholds.

8. RESULTS: Figure 4: what do the colors in the plot for the TFs mean? Please provide color scale key.

We have added in the color key at the top right of Figures 4 & 5.

*9. SUPPLEMENTAL INFO: FEATURE WEIGHT CALCULATION: 2ND PARAGRAPH: Methods 3 and 5 use a secondary PCA, not method 4.*

Thank you for catching this mistake. We have fixed it in the revision.

**Reviewer #2:**

*Integrative analyses of gene expression data from airway epithelial cells and immune cells with the airway microbiome with the objective to understand host response associated with infant RSV infection severity. The investigators used elastic net regularized regression method, which is a reasonable approach. The findings are interesting and many support prior published literature on the involvement of specific immune pathways and airway microbiome associations. The application of methods to support the integration of high dimensional multi-omics data to understand infant RSV infection severity is novel.*

We thank the reviewer for the kind comments on our work.

*MAJOR COMMENTS:
- Many of the findings support prior individual associations, which while perhaps arguing it decreases novelty, it supports the approach taken.*

Indeed, we were hearted by the fact that our novel data integration approach rediscovered some of our previous findings based on analyses of individual data types.

*- A major recognized limitation is the small sample size, but not discussed is the possibly non-random missing data.*

We have added a brief discussion of this important limitation, which we believe provides an interesting opportunity for future methods development in multi-omics data integration.

*- There is no description of cohort participants used in this analysis, specifically age of infants, time from symptom onset to collection of specimens, infant sex, antibiotic administration, and how these covariates were included in the analyses. The infant age, sex, RSV strain and time from symptom onset to collection of biospecimens could well be different based on disease severity and impact the measured host responses. Only a small subset of subjects have all data types, and there is no description of the group of 23 infants with all data types, nor the groups of 61 and 35 subjects, nor the distribution by heathcare or GRSS. It isn’t clear how or if the associations between key covariates and outcomes were included. This is particularly important for sex, infant age at time of infection, RSV strain, and time from symptom onset to sample collection.*

We have added demographic tables for the full 134 subjects (Table S1), the subset used in the nasal transcriptome, CD4 transcriptome, nasal microbiome integration model (Table S2), and the subset used in the CD4, CD8, and CD19 transcriptome integration (Table S3).

*- It is also unclear if the described 3 week follow-up biospecimens were used or could be used in these analyses to represent baseline or resolution, or if they were or could be compared with data from acute infection to assess change.*

This is outside the scope of the current manuscript; however, we previously explored this concept in [9].

*- There should be a discussion of study limitations, as well as a more robust discussion of what supports confidently knowing that L1L1 or L2L2 is the true model.*

We have added a paragraph to the Discussion describing additional limitations of this study.

While none of these models should be considered the true model, we have added an additional supplementary table (Table S4) showing performance results that justify our choice of modeling approach.

*Minor comments:
- Review articles are cited to support prior findings, and the primary source should be cited in support of specific prior findings*

We have modified the citations to address this issue.

*- A number of the individually described findings have been previously published, and these should both be recognized and those manuscripts cited*

We now cite several additional manuscripts.

*- Looking at the demographics of the study population in the referenced published methods manuscript, these aren’t “full-term”, but rather “term” infants.*

We have changed “full-term” to “term”.

*- Methods/”Weight assignment and transcription factor analysis” sub-section. The second to last sentence in this section seems to be missing a key word describing the methods.*

We thank the reviewer for catching this. It has been fixed; the sentence now reads:

“As previously described, conserved binding sites from JASPER across hg19, mm10 and rn6 were identified and were mapped to 2 kb region around Transcription Start Site (TSS) for the transcription factor analysis^37^.”

**Reviewer #3:**

*The manuscript looks original and well organized. Sometimes the reading results not fluent due to the technical details.*

We thank the Reviewer for the compliments on our work and as suggested have modified the text to increase clarity, with a focus on technical details.

*Major revisions:
1. The total number of the infant whose RT-PCR confirmed RSV illness is 139. And also, 61 subjects are in the first set and 35 in the second. Do the authors suspect that the number of patients too small? Can this small number of patients be enough to conduct an integrative analysis and deduce general results?*

It is a weakness of the study that we now discuss in the Discussion; however, we hope that the results reported herein spark additional larger scale multi-omic studies of RSV.

*2. The authors used some "preliminary exploratory analyses" to drive the research. Please, can the authors specify which kind of preliminary analyses have been conducted?*

These were primarily correlation between GRSS and individual omic features (e.g. gene expression in a particular cell type), as well as univariate regression models of GRSS as a function of different individual omic features.

*3. In the same section ("Results", lines 14-17 ), the authors used some generic words as, "relatively weak", "strong correlation", "weak features". Please, try to support with numerical evidence (tables, percentage, graphs) your assertions.*

We have added numerical evidence of these descriptions or pointed to Supplementary Figures.

*4. In the same section (Results, line 16), the authors refer to the use of an "appropriate dimension reduction". Please, can you specify which kind of dimension reduction and support your choice?*

We have clarified that we used Principal Components Analysis (PCA) for dimension reduction. This method is both the most widely used and crucially allows for the feature weighting procedure we developed in this manuscript.

*5. In the same section (Results, line 21), the authors refer to a "shared variation between data types". Please, can you document it?*

This comment is based on the results from the secondary PCA in which the secondary PCs contained primary PCs from different data types. Additionally, the number of secondary PCs was far less than the number of primary PCs from each separate data type, which also indicates shared variation. We have clarified this in the manuscript.

*5. In the section "Model application and interpretation" (line 11-24 and so on), the authors used some generic words as "clearly", "evident", "highest weighted", "unweighted", "high degree", "majority", "significantly activated", etc. Please, can the author try to support these sentences with numerical evidence?*

We have added numerical evidence to our descriptions throughout this section where possible.

*Minor revisions:
1. Supplementary figure 2. It could be better to use a different color for the column with a value of 23*

We are unsure what benefit this would provide to the reader.

*2. Section "Model application and interpretation" (line 20). Please, can the authors better specify which figure they are referring to? It is easy to be lost.*

We refer to the middle of Figure 4 and Supplementary Figures 3-12.

*3. Section "Model application and interpretation" (line 28). Please, the authors should insert the reference for the "Ingenuity" application*

We have added this citation.

*4. Section "Model application and interpretation" (line 28). The authors mention "Ingenuity suggested that ...": where are the results? In which figure?*

We refer to Figure 4 where these results can be seen.

*5. Section "Model application and interpretation" (line 35). The authors mention "upper right/Panel D": In which figure?*

We apologize for the confusion. We now refer to Figure 4 upper right.

## References

1.	Cui, Z., et al., *Super-delta2: An Enhanced Differential Expression Analysis Procedure for Multi-Group Comparisons of RNA-seq Data.* Bioinformatics, 2021.

2.	Law, C.W., et al., *voom: Precision weights unlock linear model analysis tools for RNA-seq read counts.* Genome Biol, 2014. **15**(2): p. R29.

3.	Liu, Y., J. Zhang, and X. Qiu, *Super-delta: a new differential gene expression analysis procedure with robust data normalization.* BMC Bioinformatics, 2017. **18**(1): p. 582.

4.	Rapaport, F., et al., *Comprehensive evaluation of differential gene expression analysis methods for RNA-seq data.* Genome Biol, 2013. **14**(9): p. R95.

5.	Robinson, M.D., D.J. McCarthy, and G.K. Smyth, *edgeR: a Bioconductor package for differential expression analysis of digital gene expression data.* Bioinformatics, 2010. **26**(1): p. 139-40.

6.	Misra, R.S., et al., *Flow-based sorting of neonatal lymphocyte populations for transcriptomics analysis.* J Immunol Methods, 2016. **437**: p. 13-20.

7.	Chu, C.Y., et al., *The Healthy Infant Nasal Transcriptome: A Benchmark Study.* Sci Rep, 2016. **6**: p. 33994.

8.	Chu, C.Y., et al., *Airway Gene Expression Correlates of Respiratory Syncytial Virus Disease Severity and Microbiome Composition in Infants.* J Infect Dis, 2021. **223**(9): p. 1639-1649.

9.	Wang, L., et al., *Airway gene-expression classifiers for respiratory syncytial virus (RSV) disease severity in infants.* BMC Med Genomics, 2021. **14**(1): p. 57.
