# Supplementary material for: A systems genomics approach uncovers molecular associates of RSV severity
Source: PLoS Comput Biol. 2021 Dec 28;17(12):e1009617. doi: 10.1371/journal.pcbi.1009617 (PMC8746750; doi:10.1371/journal.pcbi.1009617)
Supplement: Attachment — Submitted filename: Response to Reviewers R2.docx [file pcbi.1009617.s026.docx]

We thank the reviewers for their thoughtful comments. We have incorporated these suggestions to produce a greatly improved manuscript. Below you will find point-by-point responses. The reviewer comments are in italic.

**Reviewer #1:**

*It looks good to me.*

We thank the reviewer for their support.

**Reviewer #2:**

*The authors have been responsive to the reviewer comments and the manuscript is improved and addresses and interesting question taking a novel and challenging approach. There remains the significant limitation of the sample size and multiple testing. It would be preferable to include a statement to the methods section and the limitations section of the discussion to explain that the findings may be due to type I error due to multiple testing, this may not be obvious to all readers and would be preferable to state.*

*Suggest adding to the methods and limitations something along the lines of:
We applied a significance threshold for association using a a 2-sided p<0.05 without adjustment for multiple testing, such that findings should be considered exploratory.*

We have added the following sentence was added to the end of the Weight Assignment and Transcription factor analysis and Pathway analysis sections of the Methods: “Statistical significance was assessed at a p-value threshold of 0.05 without adjustment for multiple testing.”

And the following sentence to the Discussion: “In the pathway and transcription factors analyses, we assessed statistical significance using a p-value threshold of 0.05 without adjustment for multiple testing; therefore, these results should not be viewed as definitive.”

**Reviewer #3:**

*I wish to thank the authors for their professionality. The article appears to be ready for publication and relevant to the scientific community.*

We thank the reviewer the kind words.
